# Saturated Imaging for Inspecting Transparent Aesthetic Defects in a Polymeric Polarizer with Black and White Stripes

**DOI:** 10.3390/ma11050736

**Published:** 2018-05-07

**Authors:** Cilong Yu, Peibing Chen, Xiaopin Zhong, Xizhou Pan, Yuanlong Deng

**Affiliations:** College of Mechatronics and Control Engineering, Shenzhen University, Shenzhen 518060, China; yu@szu.edu.cn (C.Y.); chen_peibing@foxmail.com (P.C.); xzhong@szu.edu.cn (X.Z.); 2172291708@email.szu.edu.cn (X.P.)

**Keywords:** polymeric polarizer, transparent aesthetic defect, machine vision, saturated imaging

## Abstract

Machine vision systems have been widely used in industrial production lines because of their automation and contactless inspection mode. In polymeric polarizers, extremely slight transparent aesthetic defects are difficult to detect and characterize through conventional illumination. To inspect such defects rapidly and accurately, a saturated imaging technique was proposed, which innovatively uses the characteristics of saturated light in imaging by adjusting the light intensity, exposure time, and camera gain. An optical model of defect was established to explain the theory by simulation. Based on the optimum experimental conditions, active two-step scanning was conducted to demonstrate the feasibility of this detection scheme, and the proposed method was found to be efficient for real-time and in situ inspection of defects in polymer films and products.

## 1. Introduction

Machine vision refers to the efficient technology used in industrial inspection lines for automation and contactless inspection, and a variety of products require such automated inspection of defects [1,2,3]. Polymeric polarizers are crucial components of thin-film transistor liquid crystal display (LCD) panels, which are now widely used in consumer electronic products such as TVs, mobile phones, and computers. A polymeric polarizer contains six transparent polymeric film layers, and each layer might generate aesthetic defects such as bubbles, impurities, dents, stains, and scratches during the manufacturing progress. Although these defects may not affect functionality, they might be observed by end customers and may affect end user’s experience. It is thus necessary to detect defects in polymeric polarizers before packing by polarizer manufacturers and before use by LCD panel manufacturers. However, most manufacturers adopt manual inspection in a dark room; this method is labor intensive, and its detection speed and accuracy can seldom satisfy the inspection demands of modern assembly lines. Therefore, an automated inspection technology with high accuracy and speed should be developed to detect such defects for quality assurance on production lines. In particular, because of the limited resolution of the human eye, defects greater than 0.1 mm need to be detected automatically. 

Currently, few studies have focused on the inspection of defects in polymeric polarizers. Syu et al. proposed a cost-effective optical detection system for detecting tiny bump defects in polarizer films [4]. Utilizing LED illumination sources, Kim et al. designed a system for inspecting multiple defects such as bubbles, pits, threads, and alien substances, and they employed an image segmentation and template-matching algorithm to process the acquired images [5]. Chou et al. also developed a set of online real-time defect inspection systems for polymer polarizers, and complex algorithms such as down-sampling compression, wavelet transform, and Hough transform were applied to analyze the images [6]. 

The aforementioned studies have primarily inspected normal defects such as bubbles, foreign particles, marks, and scratches. However, the detection of certain special transparent defects in polymer polarizers has barely been reported. These defects, known as “convex or concave points” in the industry, have nearly identical transmissivity and reflectivity as normal regions. Thus, they have extremely low image contrast under normal illumination and are a primary problem in industrial inspection. Moreover, most studies have concentrated on image-processing algorithms; few studies have been conducted on the design of illumination sources, which is the decisive part in machine vision [7]. Various application scenarios require different lighting sources. Selecting the optimum lighting strategy greatly enhances the performance of machine vision systems and simplifies the image-processing algorithms.

We have previously implemented the inspection of the inner transparent aesthetic defects of a polymeric polarizer with black and white stripes; these special defects were barely observable when the polarizer was illuminated by a conventional white backlight. The stripes introduced the equivalent of a one-dimensional diaphragm, thereby considerably enhancing the imaging effect of the transparent defect [8,9]. When the depth of the defect and the refractive index difference (between the defect and polarizer) further decreased, the extremely slight transparent aesthetic defects (ESTADs) could not be imaged, even under structured light illumination. However, these ESTADs might be observable by end customers through edge-of-light illumination [10], thereby affecting user experience. To further a product’s retail appeal, it is necessary to check for ESTADs in polymeric polarizer. However, edge of light illumination [10] is impractical, because it is difficult to locate ESTADs at the edge of light and the image-processing algorithm costs too much time.

Hence, this paper proposes a new inspection method to detect the ESTADs using saturated imaging techniques that enhance the image contrast of transparent defects in a polymeric polarizer. Generally, saturated light reduces image quality, which is avoided when taking a picture. However, we innovatively applied the saturated imaging principle in this special defect inspection. The saturated imaging technique captures the natural divergence of light, enabling the defect to appear in the black stripe. By adjusting the light intensity, exposure time, and camera gain, the saturated effect of the camera substantially improved the contrast of the defect image, making it possible to detect ESTADs within the black stripe. Thus, the image contrast of the defect was further improved, and two steps fully covered a sample, which greatly improved the detection speed and satisfied the inspection demands of an assembly line.

## 2. Optic Simulation

**Figure 1:**
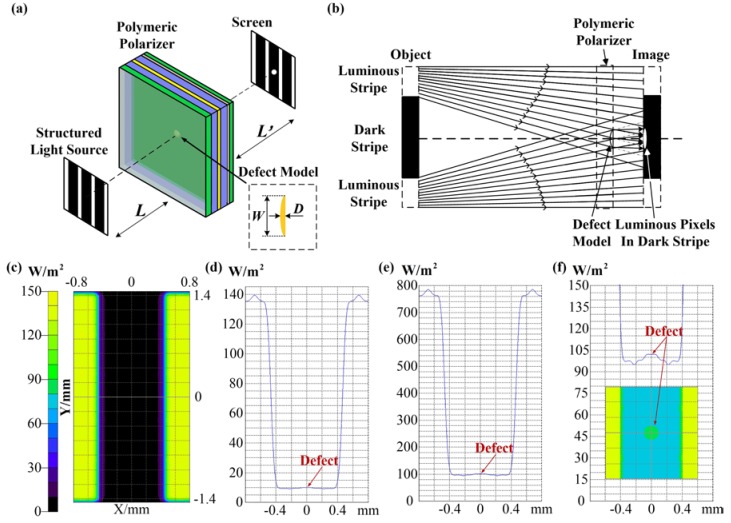
(**a**) Schematic of a TracePro simulation system; (**b**) saturated imaging mechanism for defects; (**c**) simulation result of light distribution on the screen; (**d**) light intensity along the zero axis in the horizontal directions; (**e**) higher light intensity along the zero axis in the horizontal directions; (**f**) light intensity after light intensity greater than 150 W/m^2^ was eliminated, the inserted image represents the light distribution on the screen under this condition. D = 0.01 mm, W = 0.4 mm, L = 29.846 mm, L’ = 0.923 mm.

Simple imaging theory is illustrated in Figure 1. Figure 1a presents the imaging simulation system and the optic model of defect, which are similar to those used in our previous research [8,10]. The simulation system, which used the TracePro software, contained a structured light source, a polymeric polarizer with a point defect, and a screen. The black stripe was aimed at the point defect in the polarizer, which comprised six transparent layers; the transparent defect located at the center of the second film was modeled as a microscale plano-convex lens. Because the polymeric polarizer was nearly transparent and the refractive index of the polarizer and defect were similar, this configuration (Figure 1a) is equivalent to a micro-lens imaging system without any diaphragms. Although the black stripe targeted the defect, when the white light was enhanced, the incident lights (object) from two sides of the black stripe passed through the defect (plano-convex) and then converged on a screen in the black stripe, as interpreted in Figure 1b; the defect was then imaged as a white point on the screen in the black stripe region (a set of luminous pixels in the dark stripe). Figure 1c exhibits the simulation result of light distribution illuminated at normal light intensity; the defect was barely observed on the screen. The light intensity along the zero axis in the horizontal directions was extracted from Figure 1c and is plotted in Figure 1d, the light intensity of the defect was nearly equal to that of the black strip region. When the intensity of the light source was further enhanced, the black stripe region and defect were illuminated to some extent, but the image contrast of the defect in the black stripe was barely improved, as shown in Figure 1e. A light intensity of 150 W/m^2^ corresponds to the maximum gray value, 255 in a camera system; however, light with intensity greater than 150 W/m^2^ was removed and still exhibited a gray value of 255 in the image. Figure 1f demonstrates this saturated imaging effect. Light with intensity greater than 150 W/m^2^ was eliminated, and the contrast between the defect and the black stripe was greatly enhanced. Thus, the image contrast of the defect in the black stripe region was improved using saturated imaging.

## 3. Experiment and Results

**Figure 2:**
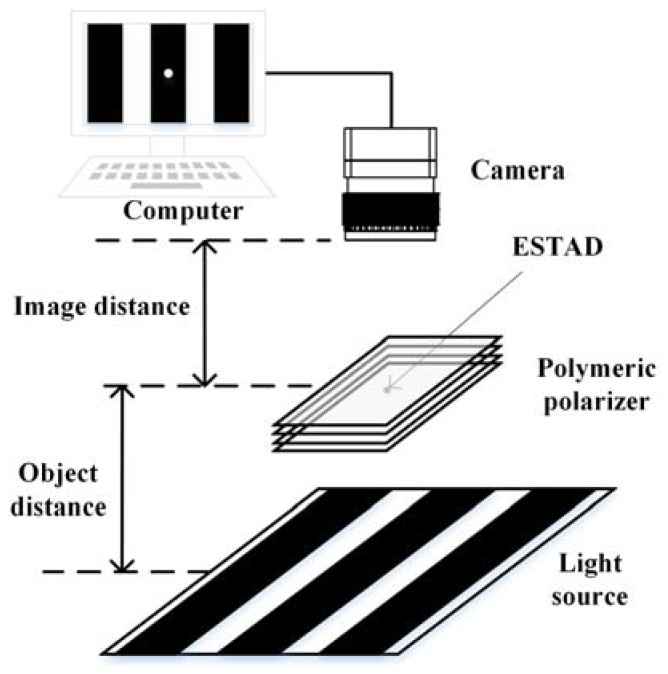
Schematic of the experimental setup.

Based on the aforementioned theory, we established the experimental system, which included a structured light source, a defective sample, and an industrial camera, as illustrated in Figure 2. A pattern of binary stripes was generated using a programmable structured light source on a tablet PC. Hence, the width, location, intensity, and shape of the stripes could be easily adjusted. The pattern was moved half of a circle of the stripe each time, and an image of the sample was then captured using the camera. Thus, it took only two steps to cover a single sample. The two static images captured in each step were processed by computer to identify defects. The setup is similar to that in our previous studies [8,10].

**Figure 3:**
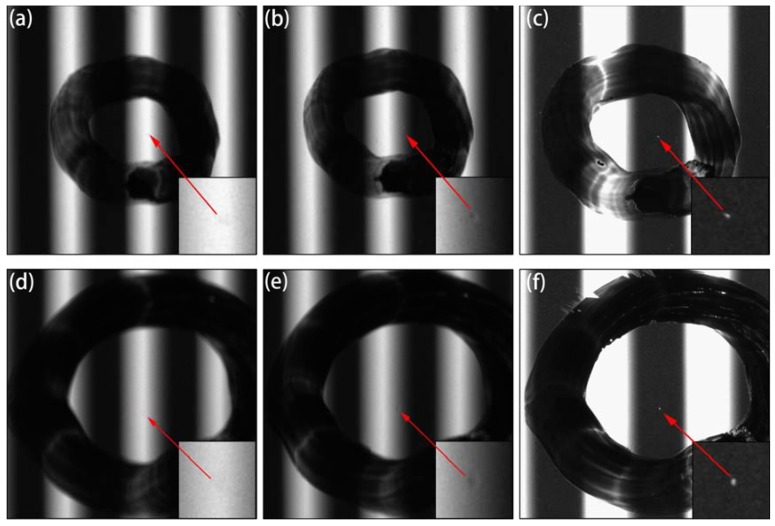
Defect images of two samples with various illumination levels: (**a**,**d**) defects with structured light illumination; (**b**,**e**) defects with edge-of-light illumination; (**c**,**f**) defects with saturated imaging technique. (**a**–**c**) were Sample 1 with a transparent impurity defect; (**d**–**f**) were Sample 2 with an indentation defect. The size of the defects was approximate 100 μm.

We have proved that special transparent defects in polymer polarizers have extremely low image contrast and are difficult to detect under uniform illumination [8]. By contrast, such defects are considerably clearer under structured light illumination. However, some ESTADs could not be imaged even under structured light illumination, as shown in Figure 3a. The application of edge of light helped to partially improve the image contrast, as demonstrated in Figure 3b, which further indicated that ESTADs might still be observed through edge-of-light illumination by end customers, thereby affecting user experience. Thus, it is necessary to inspect for ESTADs in polymeric polarizers [10]. The proposed saturated imaging technique to detect ESTADs markedly enhanced the image contrast of the defect, as shown in Figure 3c. The improvement of image contrast helped to simplify the subsequent image-processing algorithm and improve the detection speed and accuracy. 

**Figure 4:**
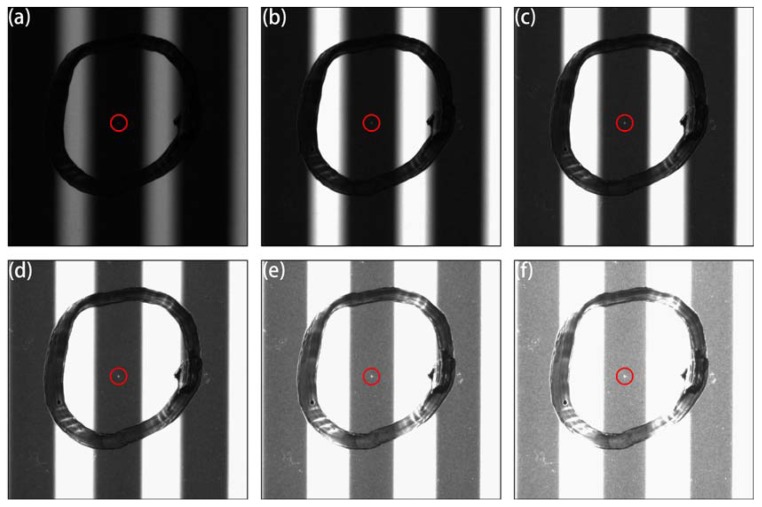
Indentation defect images with various exposure times: (**a**–**f**) 10, 30, 60, 100, 200, and 300 ms; object distance, 40 mm; image distance, 71.9 mm; camera gain, 4.25×.

Saturated imaging can be implemented in a variety of ways, such as by strengthening light intensity, increasing white stripe width, extending exposure time, and boosting gain. Figure 4 exhibits defect imaging implemented by prolonging the exposure time. When the exposure time was only 10 ms, the image was underexposed and the incident light from the white stripes barely passed through the defect, as shown in Figure 4a, and the defect could not be imaged. An increasing amount of incident light passed through the defect as the exposure time increased, which enhanced the image contrast of the defect further, achieving saturated imaging. The clearest imaging effect was at approximately 100 ms, as demonstrated in Figure 4d. The experimental images in Figure 4b,d are similar to the simulated images shown in Figure 1d,f. However, when the exposure time was prolonged further, the excess incident light bleached the black stripe region and generated granular interference points, which caused a high miss rate, as shown in Figure 4e,f. Hence, an exposure time of approximately 100 ms is optimal under such experimental conditions and should ensure high image contrast and low inspection miss rates.

**Figure 5:**
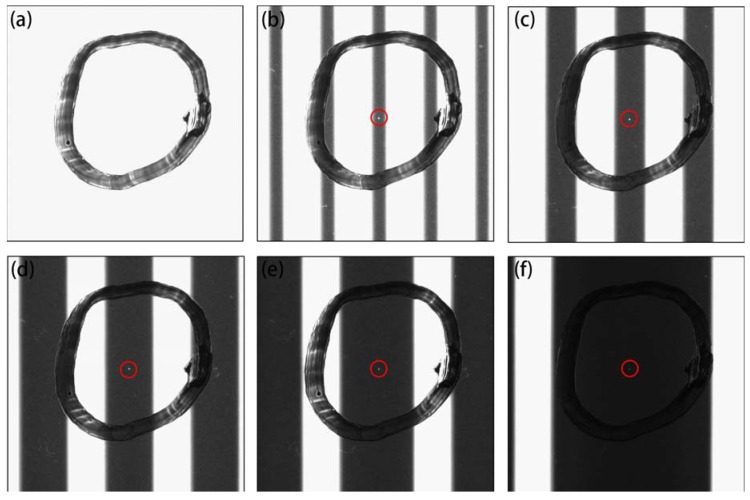
Indentation defect images with various stripe widths: (**a**) w12–b4; (**b**) w12–b12; (**c**) w12–b20; (**d**) w12–b28; (**e**) w12–b40; and (**f**) w12–b80. Object distance, 40 mm; image distance, 71.9 mm; camera gain, 4.25×; exposure time, 100 ms. The expression w12–b20 indicates that white and black stripe widths on the tablet PC were 12 and 20 pixels, respectively, whereas the exact sizes on the captured image were 2.10 and 1.58 mm, the width ratio of the black and white stripe was 0.75.

Stripe width also significantly influenced the saturated imaging process. Increasing the width of the white stripe improved the light intensity. Figure 5 demonstrates the influence of stripe width on the defect imaging. The stripe width was controlled using the programmable light source on the tablet PC. The image captured when the white and black stripe widths on the tablet PC were set as 12 and 4 pixels, respectively, is exhibited in Figure 5a; the black stripes were barely observed, which was caused by the high light intensity. When the width of the black stripe was broadened, the defect image gradually became distinct; however, the defect image disappeared as the width of the black stripe increased further. Accordingly, we drew the following three conclusions. First, if the width ratio of the black and white stripes on the captured image was less than 1, as shown in Figure 5a–c, the image contrast of the defect would be too low and a granular interference point would be produced; moreover, more scanning steps would be required to cover the whole sample, which would decrease inspection speed. Second, if the width ratio of the black and white stripes on the captured image was approximately 1–4/3, as demonstrated in Figure 5d, the defect image would be integrated and bright, making it easy to inspect. Third, if the width ratio of the black and white stripes on the captured picture was greater than 4/3, the defect image would gradually become smaller and darker until it disappeared. 

**Figure 6:**
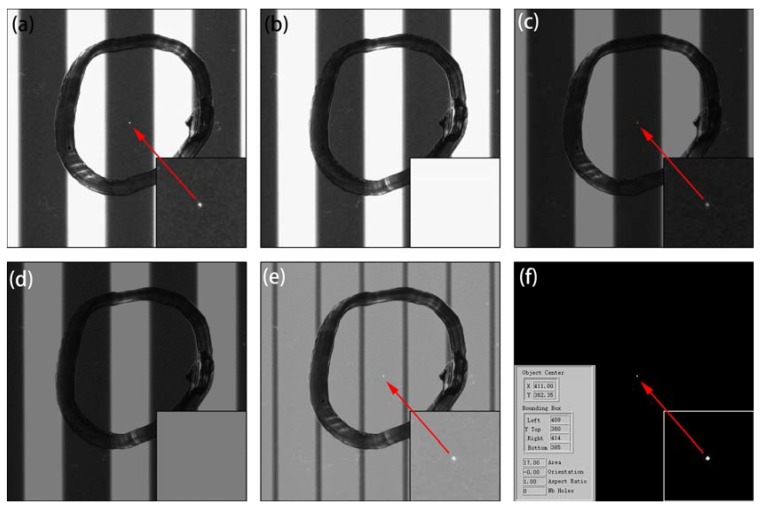
Processed images for an indentation defect sample: (**a**,**b**) sequential two-step original images; (**c**,**d**) gray value divided by 2; (**e**) superposition image; (**f**) binary image and defect extraction.

**Figure 7:**
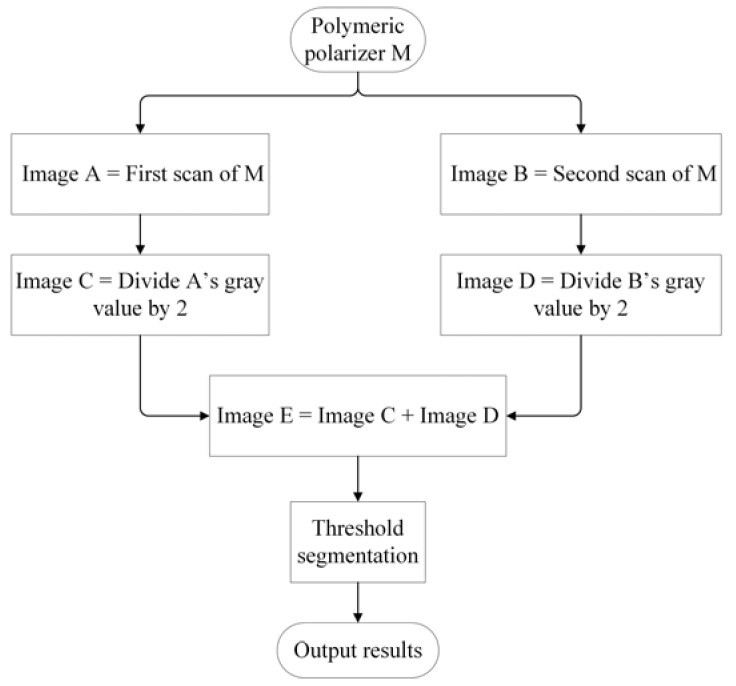
Two-step average algorithm flowchart.

Based on the aforementioned processes, active two-step scanning was performed to inspect the defect. Optimum experimental conditions were set as follows: object distance, 40 mm; image distance, 71.9 mm; camera gain, 4.25×; exposure time 100 ms; and white and black stripe widths on the tablet PC, 12 and 28 pixels, respectively. During the two scanning steps, the defect appeared on the black stripe region only once, as presented in Figure 6a, and the defect image was clear; the defect was located on the white stripe region once as well, as exhibited in Figure 6b, and it was almost impossible to discern the defect on the white stripes. Figure 6c,d exhibit the gray value of Figure 6a,b, and Figure 6e depicts the superposition of Figure 6c,d. The image in Figure 6f was obtained by binary segmentation, and the defect features, including the location, shape, and size, were extracted as shown in the inserted pictures. The whole algorithm flowchart was shown in Figure 7. This simple two-step average algorithm required only 0.51 s to execute on an ordinary PC. Because of the high image contrast, the proposed saturated imaging technique is capable of inspecting extremely slight transparent and nontransparent defects such as stains, dents, impurities, and scratches with higher speed and accuracy.

## 4. Discussion 

Simulations explored the imaging mechanism and laws of defect, though not for traditional purposes, such as specific devices or circuit designs. The parameters of the simulation shown in Figure 1 were not exactly the same as those used in the experiment, because some model parameters, such as the refractive index difference between the defect and polarizer, were approximated. It was difficult to measure the parameters precisely by experiment. Therefore, because of the parameter differences between the optic model and the practical defect, according to the lens imaging law, the values of image distance and object distance in the simulation were certainly different from that in the experiments. The specific parameter values, i.e., object distance 40 mm and image distance 71.9 mm, were decided by investigating the imaging contrast. They were also related to the camera, lens and sample. A large range of object and image distance was possible; we simply selected one group of suitable parameters in our experiments.

Several factors might affect the measurement and sensitivity. The imaging resolution determined by the camera resolution and field of vision was critical. The defect must cover at least three pixels; that is, the practical imaging resolution must exceed 33 μm/pixel. Another influential factor was the saturation degree, which was determined by the light intensity, width of black and white stripes, exposure time, and camera gain, as shown in Figure 4 and Figure 5. Excessive saturation generated granular interference points, which causes inspection mistakes, as shown in Figure 4e,f. However, if the image was underexposed, the defect could not be seen, which causes defects to be overlooked at a high rate, as shown in Figure 4a,b. It was also necessary to adjust the imaging parameters, such as object and image distance, to obtain the optimum defect imaging effect. Apart from these main parameters, some general factors have also been introduced in references [8,9,10].

A previous study [10] discovered that ESTADs are difficult to detect. We tested optic models of ESTADs through simulation and experiments, and found that although ESTADs were transparent and diminutive, they still might be observable by end customers through edge of light illumination and thereby affect the quality of products. Thus, it is necessary to inspect for these ESTADs in polymeric polarizers. However, edge of light illumination lacks practicality, because it is difficult to locate the ESTADs at the edge of light, and the image-processing algorithm required approximately 90 s to identify ESTADs. Therefore, this study proposed a new method, which utilized the natural divergency of light, enabling the defect to appear in a black stripe. Combining the saturated effect of a camera, as shown in Figure 1, the image contrast was improved greatly, making it possible to identify ESTADs in the black stripe. The new imaging mechanism and inspection method are the novelties of this paper; the contribution was not simply to optimize parameters based on previous reference [10]. Simulation results were consistent with the experimental results, which verified the imaging theory. Moreover, only 0.51 s was required to perform the simple two-step average algorithm on an ordinary PC. Experiments demonstrated the practical feasibility of this saturated imaging technique. Although this paper concerns the detection of ESTADs, the proposed method is also suitable for inspecting various cosmetic defects, such as stains, dents, impurities, and scratches. All the defect samples (approximately 100) in our lab were inspected correctly without missing and erroneous inspection.

## 5. Conclusions

A new method of saturated imaging was first proposed for inspecting ESTADs that are difficult to inspect due to their nearly identical transmissivity and reflectivity as those of normal regions. The presented method effectively increased the image contrast, which greatly simplified the image-processing algorithm and improved the inspection accuracy and speed. 

An optical defect model and the saturated imaging theory were established in this paper, which were benefit to understand the imaging mechanism, and also demonstrate the possibility of this method. Experiments with various illumination conditions were performed to prove that this saturated imaging technique drastically enhanced the image contrast of the defect. The influence of exposure time and the stripe width on saturated imaging were explored, specifically, the optimum exposure time was 100 ms, and the white and black stripe widths were 12 and 28 pixels respectively in this detection system. The active two-step scanning method was applied to scan the whole sample and a simple two-step average algorithm was proposed to extract the defect. Each inspection took only 0.51 s on an ordinary PC and so far the inspection accuracy was 100%. Thus, this method appears to be efficient for real-time and in situ inspection of aesthetic defects in polymer films in industry. Furthermore, the machine learning algorithm might be introduced to identify and classify various defects in the future work.
